# The Impact of Perceived Stress and Coping Adequacy on the Health of Nurses: A Pilot Investigation

**DOI:** 10.1155/2016/5843256

**Published:** 2016-11-01

**Authors:** Timothy R. Jordan, Jagdish Khubchandani, Michael Wiblishauser

**Affiliations:** ^1^The University of Toledo, Toledo, OH 43606, USA; ^2^Ball State University, Muncie, IN 47306, USA; ^3^Department of Health Sciences, Lock Haven University, Lock Haven, PA 17745, USA

## Abstract

Stress and coping abilities influence the health and work performance of nurses. However, little is known about the combined influence of stress perception and perceived coping adequacy and its impact on the health of nurses. This study examined the relationship between stress, coping, and the combined influences of perceived stress and coping abilities on health and work performance. A valid and reliable questionnaire was completed by 120 nurses in a Midwestern hospital in the USA. In general, the nurses were not healthy: 92% had moderate-to-very high stress levels; 78% slept less than 8 hours of sleep per night; 69% did not exercise regularly; 63% consumed less than 5 servings of fruits and vegetables per day; and 22% were classified as binge drinkers. When confronted with workplace stress, 70% of nurses reported that they consumed more junk food and 63% reported that they consumed more food than usual as a way of coping. Nurses in the “high stress/poor coping” group had the poorest health outcomes and highest health risk behaviors compared to those in other groups. The combined variables of perceived stress and perceived coping adequacy influenced the health of nurses. Therefore, worksite health promotion programs for nurses should focus equally on stress reduction, stress management, and the development of healthy coping skills.

## 1. Introduction

(*1) Background*. Nurses make up one of the largest segments of the global healthcare workforce. On a daily basis, many nurses work on the front lines providing care to patients while serving as a lifeline of information, encouragement, and education to family members of patients. Hence, keeping nurses healthy and productive is an obvious priority for health care systems [1–5].

Because nurses are well educated in the health field, one would naturally assume that their health status and health behaviors would be better than the health behaviors of the general population. However, past research has indicated that many nurses are not healthy. For example, Zapka and colleagues reported in their study that 13.6% of nurses had hypertension, 21.5% had high cholesterol levels, 65.4% had body mass indexe higher than 25, and most consumed an average of 4 servings of fruits and vegetables per day while eating diets high in fats. Furthermore, more than a third of nurses do not exercise regularly and more than 1 in 10 may be using tobacco according to various estimates [1–3].

The challenges that nurses face in practicing healthy behaviors are exacerbated by the fact that nurses have many competing demands for their time, energy, and attention. Nurses must focus on the health of their patients, the needs of patients' family members, the demands of physicians and supervisors, their own needs, and the concerns of their own family members. The increasing demands faced by nurses impact all areas of nurses' personal and professional lives and increase their risks of chronic stress, work-family conflict, and unhealthy behaviors [4–6]. In addition, the long work hours and unconventional work schedules in the nursing profession often contribute to nurses' feelings of being overworked [5–8].

(*2) Stress among Nurses and Its Impact on Health.* Within a hospital setting, nurses often face multiple sources of work-related stress including constant noise, interpersonal conflicts with other healthcare professionals, workload demands, conflicts with physicians, role conflicts, dealing with death and dying, lack of resources, lack of support from coworkers and supervisors, patient aggressiveness or violence, increasing patient loads, and challenging patients [8–11]. The social environment of the workplace should not be underestimated in its ability to impact the stress level and health status of employees [9–14]. For example, verbal abuse or harassment from supervisors, from coworkers, or from patients may lead to negative emotional coping behaviors (e.g., anger, humiliation, shame, and frustration) and negative physical health symptoms (e.g., stomach pain, headaches, and difficulty in sleeping). Nurses who are bullied or harassed may develop emotional problems (e.g., mood swings, anxiety, depression, and fear) or psychosomatic related health problems (e.g., gastric problems, headaches, and sensitivity to sounds) in as little as a few months of working in a negative work environment [6–14].

The very nature of nurses' work can also induce stress. Nurses often experience low levels of autonomy and low control over their job, are moved among different patient care units, experience poor communication among members of the health care team, and often deal with demanding and/or uncooperative family members of patients. Such work-related stressors increase nurses' feelings and perceptions of being overworked and stressed [8–12].

The association between stress and negative health outcomes is well documented. Stress can alter human homeostasis and physiological or hormonal balance, changed that are highly associated with health problems [9–11, 15–17]. Furthermore, past longitudinal research with nurses has documented a robust association between stress and chronic diseases such as cancer and mental health problems [3, 4, 9]. Chronic work-related stress has been identified as a contributing factor to nurses' negative health behaviors including unhealthy eating habits, lack of physical activity, and ATOD (alcohol, tobacco, other drugs) use [1–3, 18–20]. 

(*3) Methods of Coping with Stress and Self-Efficacy.* It is impossible to remove all stress from the work life of nurses. Therefore, it is important for nurses (and their employers) to find healthy ways for nurses to cope with work-related stress. The effectiveness of employees' coping techniques affects their health and well-being [18–21]. Nurses who turn to unhealthy coping methods often use food, use ATOD (alcohol, tobacco, and other drugs), or develop sedentary lifestyles as a byproduct of the negative coping techniques (e.g., binge TV watching) [1–3, 8–10].

A high level of self-efficacy can help nurses more effectively cope with work-related stress. Past research has demonstrated that higher levels of self-efficacy coupled with social support facilitate healthier lifestyles and healthier coping behaviors for employees in high stress professions such as nursing [15–21]. Self-efficacy is defined as a cognitive attribute that helps to determine how well a person can organize and execute behaviors required to deal with prospective situations containing many ambiguous, unpredictable, and often stressful elements. An individual's decision to engage in and persist in performing a specific behavior or task is dependent on his/her level of perceived self-efficacy which in turn influences one's choices of activities, the amount of effort he/she will expend, and how much time he/she will invest in the task, despite hurdles, opposition, and challenges [15–19]. Therefore, nurses with higher levels of coping self-efficacy to cope with professional challenges are more likely to stay in the nursing profession, work hard while on the job, and perform their job tasks effectively, even in the midst of challenges and high stress [1–9].

(*4) Aims of This Study.* Although there is ample published evidence on the causes of stress in nurses and the impact of stress on their health, little is known about the combined impact of perceived stress and perceived coping adequacy on the health status and health behaviors of nurses. Therefore, the aims of the current study were to assess nurses' health status, health behaviors, self-reported stress levels, coping techniques, perceived coping effectiveness, and situation specific self-efficacy to cope with workplace related stress. More specifically, we wanted to further elucidate the relationship between stress, coping adequacy, and health.

## 2. Methods

### 2.1. Study Design and Participants

The study was a cross-sectional observational study that was designed based on best practices in survey research. Participants for the study included all full-time and part-time nurses employed by a community hospital in the Midwestern United States (*N* = 177). Approval was obtained from the hospital's Institutional Review Board (IRB)/Human Subjects' Committee before starting the study.

### 2.2. Survey Instrument Development and Pilot Testing

The investigators designed a valid and reliable survey instrument to measure nurses' self-reported health status, current health behaviors, history of preventive medical behaviors, level of work-related stress, self-efficacy of coping techniques, and negative manifestations of stress. Face validity of the instrument was established by basing the content of the survey on a comprehensive review of the published research literature regarding the nursing profession, health behaviors of nurses, stress, and coping self-efficacy. Face validity was further established by using validated and reliable items and subscales from previously published research (e.g., items from the Behavioral Risk Factor Surveillance System and the PHQ-4 [18, 19, 22, 23]).

Content validity of the survey instrument was developed by seeking feedback from an expert panel of six nurses and nurse administrators with many years of experience in the nursing profession. Recommended edits from this expert panel were incorporated into the survey prior to pilot testing.

After revising the survey, the investigators established the stability-reliability and internal reliability of the instrument via pilot testing. Stability-reliability of the survey instrument was assessed by collecting data from a sister hospital in the same city where a random-sample of 27 nurses were asked to complete the survey twice within a period of 10 days. Intraclass correlation analyses were used for survey items that featured continuous data. Kappa statistics (%) were used for items that featured categorical data. The stability-reliability coefficients for the various sections of the survey ranged from 0.63 to 0.88. Internal reliability coefficients (Cronbach's Alpha) for the primary subscales of the survey instrument were assessed from the final study sample (*n* = 120) and were as follows: Manifestations of Stress assessed by PHQ-4 [(4 items) Cronbach's Alpha = 0.79], Frequency of Coping Mechanisms [(8 items) Cronbach's Alpha= 0.72], and Self-Efficacy of Coping with Stress [(15 items) Cronbach's Alpha = 0.89].

After final revisions, the survey consisted of 65 items organized into a 4-page booklet style document. The first page of the survey consisted of 23 items. Part A of the survey consisted of 8 items that assessed nurses' health status, including how many days in the last month they had experienced various positive and negative manifestations of health. Part B of the survey assessed nurses' health behaviors including exercise, diet, sleep patterns, tobacco use, and alcohol use. Part C of the survey assessed nurses' prevention/screening history including history of mammography, PAP, colonoscopy, blood sugar, blood lipids, blood pressure, general physical exam, and PSA testing.

The second page of the survey (Part D) focused on work-related stress. Nurses were asked to rate their level of work-related stress at the hospital; rate how well they deal with work-related stress; and identify the techniques that they use to cope with stress. Nurses were also asked to indicate how frequently in the last two weeks they had experienced specific negative manifestations of stress (i.e., symptoms of anxiety and depression). The PHQ-4, a brief screening measure for depression and anxiety, was used in the questionnaire. This four-item valid and reliable measure to screen for depression (2 questions called PHQ-2) and anxiety (2 questions called GAD-2) was used due to high sensitivity and brevity of the measure [22].

Page 3 of the survey was comprised of three major sections that dealt with the following mental and physical health areas: perceived self-efficacy to cope with specific, potentially stressful situations at work, as well as eating habits at work. Page 4 of the survey included sociodemographic variables such as sex, age, race, marital status, educational level, and credentials.

### 2.3. Data Collection

Following IRB approval, a three-wave postal mailing procedure was used to mail surveys to nurses' homes. The first wave consisted of a written survey instrument, a personalized, signed letter of invitation, a self-addressed, stamped return envelope, and a $1 bill. The survey instrument was printed as a four-page booklet on pastel green paper. The personalized letter of invitation described the purpose of the study and stressed the importance of completing the survey. The second wave was mailed approximately two weeks after the first wave and consisted of another copy of the survey instrument, a personalized, signed reminder letter of invitation, and a self-addressed, stamped return envelope. The third wave consisted of a postcard reminder mailed approximately two weeks after the second wave. These procedures that have been frequently used with health professionals were employed to maximize the response rate, thus reducing the threat to external validity [24, 25].

### 2.4. Data Analysis

The data from the completed surveys were analyzed using SPSS for Windows Version 22.0. Descriptive statistics (e.g., frequencies, mean, and standard deviation) were used to describe the respondents and their responses on various survey items. To test for statistically significant group differences, we used Chi-Square tests for categorical variables and ANOVA or *t*-tests for continuous variables. A new variable was created based on the perceived stress and coping ability of study participants and the entire population was categorized into 3 groups based on various combinations of perceived stress and perceived coping ability. Statistical significance was determined a priori at *P* < 0.05.

## 3. Results

### 3.1. Description of Respondents

Of the 177 surveys mailed, two nurses refused to answer the survey (i.e., voiced their objections via telephone) and one survey was returned as undeliverable. Of the remaining 174 surveys, a total of 120 nurses responded by completing the survey (69% return rate). The respondents can be described as female (96%), Caucasian (95%), day shift workers (71%), full time (68%), and married (68%). The mean age of respondents was 41 years (SD = 10.8). Respondents had been working as nurses for an average of 15 years (SD = 11.9). The average time of working at the hospital was five years (SD = 5.6). A plurality of the registered nurse respondents had 2-year degrees (38%) and worked in the Medical/Surgical Department (22%).

The average body weight of the nurse respondents was 168 lbs. (SD = 41.31). The average Body Mass Index (BMI) for respondents was 27.92, meaning that the average respondent was classified as “overweight.” Interestingly, full-time nurses were more likely to have statistically significant higher body weight (mean = 173 pounds) than part-time nurses (mean = 155 pounds) [*t* = 2.61, df = 118, and *P* = 0.01]. Similarly, night shift nurses had higher body weight (mean = 171 pounds) compared to day shift nurses (mean = 166 pounds). However, the body weight differences between night shift and day shift nurses were not statistically significant. Nurses who worked 12-hour shifts were more likely to have statistically significantly higher body weight (mean = 174 pounds) than nurses who usually work 8-hour shifts (mean = 158 pounds) [*t* = −2.12, df = 108, and *P* = 0.03]. Nurses who worked in Medical/Surgical Department or ICU were more likely to have statistically significantly higher body weight (mean = 182 pounds) compared to nurses that work in other departments (mean = 162 pounds) [*t* = 2.42, df = 117, and *P* = 0.02].

### 3.2. Stress Levels

Nurses were asked to rate the level of stress that they were currently experiencing at work. The vast majority of nurses (67%) rated their stress levels as “moderate.” An additional 25% rated their stress level as “high” to “very high.” Thus, 92% of nurses rated their stress level in the “moderate” to “very high” range (Table 1).

Nurses' self-reported stress levels were analyzed to determine if there were statistically significant differences across selected sociodemographic variables such as age, race, full-time versus part-time status, night shift versus day shift, length of shift, and department. Nurses who worked full time were more likely to have “very high” or “high” stress (31%) compared to those who work part time (11%) (*χ* ^2^ = 4.92, *P* = 0.02). Nurses who worked in the Medical/Surgical Department or ICU were more likely to have very high or high stress (39%) as compared to those who work in other departments (18%) (*χ* ^2^ = 6.25, *P* = 0.01).

### 3.3. Coping Methods and Coping Adequacy

Nurses were asked to select from list of 13 common methods of coping with stress. The five most common ways that nurses cope with work-related stress were as follows: talking with friends and loved ones (79%); listening to music (46%); watching TV (43%); praying/meditating (43%); and eating more of their favorite foods (42%) (Table 1).

Nurses were asked to rate how well they typically cope with work-related stress. In general, nurses believed that they were coping well with work-related stress. The majority (71%) reported dealing with work-related stress “*well*” or “*very well*.” In contrast, only 4% of nurses believed they were dealing with stress “*poorly*” or “*very poorly*.” Nearly one in four nurses (24%) believed that their coping skills were neither good nor bad, thus indicating that there is room for improvement in their coping skills (Table 1).

We analyzed the nurses' self-reported coping ability (i.e., perceived coping adequacy) to determine if there were statistically significant differences across selected sociodemographic variables such as age, race, full-time versus part-time status, night shift versus day shift, length of shift, and department. No statistically significant differences in self-reported coping ability were detected across these variables.

In terms of the impact of work-related stress on nurses' health behaviors, 70% of nurses reported that they “*sometimes*” to “*every time*” consumed more junk food when confronted with work-related stress. Similarly, 63% of nurses reported that they “*sometimes*” to “*every time*” consumed more food than usual as a way of coping with stress. More than half (61%) of nurses “*sometimes*” to “*every time*” slept less than usual when confronted with work-related stress (Table 2).

As described previously, a five-point Likert scale assessed perceptions of worksite stress (very high to very low) and ability to cope with stress (very well to very poorly). These two variables were combined to categorize nurses into three groups: (1) high stress and poor coping, (2) high stress or poor coping, (3) low stress and good coping. We compared these 3 groups for various health risk factors and health outcomes [15–21].

### 3.4. Health Status

The vast majority of respondents rated their health as “very good” (58%) to “excellent” (10%). Almost a third of the participants rated their health as “good” or “fair” (32.5%). Respondents were asked to identify from a list how many days in the last 30 days they had experienced various health manifestations or symptoms. In terms of positive health manifestations, nurses reported feeling healthy and full of energy less than 50% of the time—an average of only 14.48 days out of the last 30 days (SE = 0.99). Nurses reported feeling sleep deprived an average of 12.30 days out of the last 30 days (SE = 0.78). Mental health and dietary health were also identified as negative health issues. Nurses reported feeling worried, tense, or anxious an average of 8.48 days out of the last 30 days (SE = 0.89). Moreover, nurses reported eating too much on an average of 8.24 days out of the last 30 days (SE = 0.86) (Table 3).

We analyzed the nurses' self-reported health status to determine if there were statistically significant differences across selected sociodemographic variables such as age, race, full-time versus part-time status, night shift versus day shift, length of shift, and department. Nurses who worked 8-hour shifts were more likely to report “very good” or “excellent” health status (83%) compared to nurses who worked 12-hour shifts (61%) (*χ* ^2^ = 5.77, *P* = 0.01). Likewise, nurses who worked 8-hour shifts reported feeling healthy and full of energy on more days compared to nurses who worked 12-hour shifts. Nurses who worked 8-hour shifts (M = 17.47, SE = ±1.45) reported feeling healthy and full of energy an average of 7 more days in the last month compared to nurses who worked 12-hour shifts (M = 10.03, SE = ±1.30, *t* = 4.15, df = 117, and *P* < 0.001).

Health status in the past month was compared across the three categories of stress and coping. Nurses who had high stress and poor coping had statistically significantly higher days of feeling tense, worried, or anxious, suffering from pain, feeling depressed and sad, and inadequate sleep (Table 3).

### 3.5. Health Behaviors

In general, the nurses were not a healthy population in terms of their health behaviors. Nearly 4 of 5 nurses (78%) got less than 8 hours of sleep per night. The vast majority (68% to 69%) do not exercise regularly. Nearly 2 of 3 nurses (63%) did not eat 5 or more servings of fruits and vegetables per day. More than 1 in 5 nurses (22%) were classified as binge drinkers and 14% used tobacco in the 30 days preceding the survey (Table 4). Across the 3 categories of individuals (based on stress and coping), the nurses who had high stress and poor coping were more likely to use tobacco, more likely to consume more than 5 alcoholic drinks on any occasion, less likely to consume adequate fruits and vegetables, and less likely to get adequate sleep. Nurses with low stress and good coping skills had a higher likelihood for engaging in all of the selected healthy behaviors (Table 4).

### 3.6. Stress Manifestations: Depression and Anxiety Symptoms

We asked nurses to rate how often they had been bothered by common depression and anxiety related symptoms in the last two weeks prior to completing the survey by using the PHQ-4 screening tool [22]. Each question of PHQ-4 had potential response options (scored 0 to 3 indicating low-to-high frequency of symptoms) (Table 5). The most commonly reported symptom was* “feeling nervous, anxious, or on edge.”* More than half (52%) reported feeling this way for several days in the last two weeks. Furthermore, almost 1 in 5 nurses (17%) reported feeling this way on more than* “half the days”* or* “nearly every day”* in the last two weeks. In contrast, only 4% of nurses reported* “feeling down, depressed, or hopeless”* or* “experiencing little interest or pleasure in doing things.”* The total score on PHQ-4 for each individual can range from 0 to 12 [22]. Across the 3 categories of nurses (based on stress and coping), the nurses who had high stress and poor coping were more likely to have high scores on the PHQ-4 depression and anxiety screening scale. For two of the four depression/anxiety symptoms, the group with good coping and low stress had statistically significantly lower scores among all groups (Table 5).

### 3.7. Preventive Health and Screening Behaviors

In the area of preventive screening behaviors, the majority of the nurses had not completed a mammogram, blood sugar profile, or blood lipid profile in the last 1 year. Across the 3 categories of nurses (based on stress and coping), the nurses who had high stress and poor coping were the least likely to have preventive screenings completed compared to groups with low stress and good coping. These differences were also statistically significant for some preventive actions (Table 6).

### 3.8. Barriers to Healthy Eating

Regarding daily diet, a significant proportion of nurses (47%) reported that they ate less healthy food at work than they do at home. However, a plurality also suggested that their diet is basically the same whether at home or at work (41%). Few nurses reported eating less healthy at home than at work (12%). Participating nurses were presented with a list of common barriers to healthy eating at work and asked to identify the barriers to healthy eating at their hospital. The nurses identified the following barriers: limited choices of healthy foods at work (60%), too much work to do during their shift (33%), cafeteria not being open during their shift (30%), poor quality of food at this hospital (21.7%), cost of healthy food being too high (20.8%), taking too long to go find healthy food (20%), and not enough time to eat healthy (18.3%). A barrier score was created by adding all the individual barriers to eating healthy. Each barrier was assigned a score of 1. The potential range of the barrier score was 0 to 7. On average, all nurses faced two or more barriers to eating healthy (M = 2.63, SE = ±0.45). By using an ANOVA test, we compared nurses' barrier scores across the 3 categories of stress and coping. Nurses with high stress* and* poor coping (M = 3.08, SE = ±0.69) reported statistically significantly more barriers to eating healthy than nurses with high stress* or* poor coping (M = 2.25, SE = ±0.41) and low stress and good coping (M = 1.88, SE = ±0.36) (*F* = 4.50, *P* = 0.04).

### 3.9. Situation Specific Self-Efficacy

Nurses were asked to rate how confident they were in coping with common situations in the clinical setting that are often viewed as stressful situations. The proportion of nurses that reported the highest levels of coping self-efficacy (i.e., felt “confident” to “very confident” range) were as follows: handling difficulties with patients (92%); getting work done during one's shift (91%); handling difficulties with patients' family members (88%); coping with medication errors made by others (87%); delegating tasks to ancillary staff (84%); and having to do a lot of tasks at the same time (84%) (Table 7). In contrast, a significant proportion of nurses reported low levels of coping self-efficacy (i.e., ratings of “not sure of my confidence,” “minimal confidence,” or “no confidence at all”) when dealing with the following situations: poorly defined or unclear procedures (33%); medication errors that they make (30%); lack of timely follow-through by support staff (26%); equipment that is malfunctioning (26%); relational difficulties with colleagues (25%); and relational issues with physicians (25%) (Table 7).

Situation specific confidence was assessed across the 3 categories of nurses (based on stress and coping) (Table 7). Nurses with low stress and good coping had the highest confidence in dealing with patient difficulties, getting work done during the shift, having to do multiple tasks at the same time, delegating work, dealing with lack of timely follow-through by support staff, managing difficulties with supervisor, and solving relational issues with physicians. In contrast, nurses with poor coping and high stress had low confidence in dealing with these same situations.

## 4. Discussion

The aims of the current study were to delineate the relationship between nurses' stress, coping adequacy, and health and work performance by assessing their health status, health behaviors, self-reported stress levels, coping techniques, perceived coping effectiveness, and situation specific self-efficacy to cope with workplace related stress. Overall, the nurses rated themselves as healthy, with majority of them rating their health as very good or excellent. Yet, nurses reported feeling healthy and full of energy less than 50% of the time during the 30 days preceding the survey. Furthermore, inadequate sleep was a common problem with nearly 4 of 5 nurses sleeping less than 8 hours per night on an average day.

Although the majority of nurses rated their health as good, there was an obvious disconnect between nurses' health status and their health behaviors. The vast majority did not exercise regularly and did not eat the recommended number of daily servings of fruits and vegetables. This was clearly evidenced by the proportion of nurses who were overweight. Furthermore, we found that binge drinking and tobacco use were risk factors in this population. These findings are similar to other studies with healthcare professionals across various geographic locations [5–8].

Interesting differences in health status were noted by the nurses' typical shift, duration of shifts, work status category (full time versus part time), and department. For example, nurses who worked 8-hour shifts weighed an average of 16 pounds* less* than nurses who worked 12-hour shifts. In other professions, these findings have been confirmed across many studies. It was interesting to note that body weights of nurses differed significantly by work status and department. Full-time nurses carried approximately 20 more pounds of body weight than part-time nurses. Nurses who worked in Medical/Surgical Department or ICU carried an average of 20 more pounds of body weight than nurses who worked in other departments. Nurses' body weights are likely influenced by their eating habits at work. Two of five nurses reported that they eat less healthy at work than they do at home. Barriers to healthy eating at work included limited choices of healthy foods, too much work to do during their shift (not enough time to eat healthy) and, the cafeteria being closed during their shift. Eating habits of nurses were also influenced by stress. The vast majority of nurses reported that they often consume junk food when confronted with work-related stress. Furthermore, eating more food than usual was a common coping technique for nurses when confronted with work-related stress. Again, these findings mirror the findings from workers in other professions and in the general population [5–8, 18, 19].

Stress and coping efficacy independently affect the health of an individual. Stress can challenge the normal physiology and hormonal balance of individuals and the way an individual may cope with stress would define the health outcomes for the individual [26–30]. The predominant focus of this study was the combined effects of stress and coping on health and work performance. One of the most interesting findings of this study was that more than 90% of nurses rated their stress levels at work in the “moderate” to “very high” range with higher level of stress in full-time nurses and those who worked in Medical/Surgical Department or ICU. Although stress was present in the workplace, the majority of nurses reported dealing with work-related stress “*well*” or “*very well.*” Given this interesting combination, our analyses were unique because we assessed the differences between nurses based on three permutations and combinations of perceived stress levels and coping with stress (i.e., group 1 = high stress and poor coping, group 2 = high stress or poor coping, and group 3 = good coping and low stress). Our assumption that both stress and coping jointly influence health, well-being, and work performance of nurses was illustrated by the results of our analyses. Nurses in group 1 had the highest health risk behaviors, the poorest health status, and the least confidence in dealing with workplace assignments that could be stressful (Table 7). Lazarus and colleagues, the pioneers in the field of stress and coping, and several others have reported that stress alone cannot but excess and chronic stress that overextends the individual's ability to cope with their challenges can result in undesirable health outcomes [26–30]. Also, from the employers' perspective, this suggests that effective workplace health promotion programs should focus on stress management and stress reduction in addition to teaching employees the skills of coping [8–10, 18, 19]. Moreover, the skills of healthy coping need to be strengthened through repeated guided practice and positive reinforcement [26–31].

Workplace stress is becoming a normal phenomenon in modern societies. However, a high stress situation may not necessarily be detrimental to the employee as long as he/she has learned to cope with it in a healthy manner. To this end, employers and workplace health promotion specialists can implement an array of employee wellness activities to assist nurses with learning how to recognize and cope with stress in a healthy way [18–21, 27–33]. Many companies and businesses have implemented specific programs to decrease absenteeism and loss of work productivity due to illnesses among their employees [32, 33]. Wellness programs, which are created to promote healthy living habits, have been shown to improve workers' health, decrease worker absenteeism, and increase job satisfaction [18, 19]. Also, some worksites have offered their employees programs such as smoking cessation programs, programs designed to reduce cardiac risk factors, programs to prevent and reduce mental health issues in employees, and programs designed to increase physical activity [31–33]. There is ample proof that evidence-based, health promotion and disease prevention practices in the workplace improve employee health and well-being. If hospitals have the social, political, and economic will, they can invest in initiatives to improve health and work performance of nurses by targeting major stressors [31–33]. Also, much of the social and professional environment at work is created by supervisors, administrators, and workplace policies [5–8]. The role and importance of nursing supervisors/administrators cannot be overstated. Good leadership and management skills exhibited by nursing supervisors can act as buffers and help nurses to cope with stress in healthy ways. In contrast, when nurses perceive problems with the quality of leadership or when supervisors fail to address work-related problems, stress levels and feelings of being overworked are exacerbated [6–14].

In addition to the aforementioned practice recommendations, there are policy, education, and research implications as well for nurses and the influence of worksite stress on health [8–12, 15–18, 31–33]. First, in relation to policymaking, hospitals and healthcare facilities need to have robust policies that enable health promotion and better physical and structural working avenues. At the organizational level, there should be reasonable staffing practices, flexible scheduling, good communication practices and structures, interactive and transparent labor and management relations, a culture of continuous support and learning given the emotional and role demands, better job security, structuring of team relationships and role clarity, and provision of greater occupational health and safety. Also, policies on tobacco use, the food available in cafeterias, the business hours for the cafeteria, the foods and beverages offered in vending machines, availability of fitness centers, walking paths, employee assistance programs, health benefits like insurance, free annual checkups, and incentives for maintaining a healthy lifestyle, and so forth can have a profound influence on health of nurses even if they have a stressful daily routine. In the long run, hospitals can have greater return on investment by keeping their nurses healthy by implementing policies and systems that facilitate health promotion and disease prevention. Second, education of nurses is another avenue to improve their health and wellness. Educational interventions should include a variety of group, individual, or web-based learning activities and problem-based educational strategies to promote learning about personal health, coping with stress, ensuring personal safety, self-management of diseases, maintaining work-family balance, and dealing with complex professional practice situations that eventually affect health and work performance. Finally, even though a lot has been published on nurses' health across the world, there is a need for effective evidence-based practices to improve health and well-being of nursing workforce in a variety of geographic settings and across different cultures. Health promotion researchers should actively collaborate with nurses, hospital managements, and healthcare partners to design, implement, and evaluate the effectiveness of interventions aimed at improving nurses' health, safety, and well-being using rigorous research and evaluation methodologies [3–12].

Our study provides a reasonable example of a baseline assessment of nurses' health in a hospital setting. The next logical steps would include prioritizing needs, conceptualizing health promotion programs, identifying and implementing the best practices, and conducting periodic evaluation of the effectiveness of the health promotion initiatives for nursing staff. The costs involved in such employee wellness activities would likely be far less than the costs associated with ignoring the health and wellness of nurses. Nurses with stress-induced absenteeism, mental health problems, and physical health problems cost hospitals far more than healthy nurses. Healthy nurses are also more likely to provide better nursing care [12–19].

The results of this study should be interpreted with potential limitations in mind. First, this is a small study of one hospital in the Midwestern United States. The small sample size limits the statistical power of the study. The small size and homogeneous nature of the sample also limits the external validity of the study. Second, the data were self-reported. Although careful steps were taken to protect the confidentiality and anonymity of respondents, some may have believed that their survey responses would be seen by their superiors. Thus a response bias may have occurred due to nurses reporting more positive (i.e., socially desirable) answers, particularly on items dealing with personal health habits (e.g., substance use.) Therefore, the actual health habits of nurses may be different from what was reported. Third, the data were cross-sectional in nature. The results are but a one-time “snap shot” which can only be used to evaluate the sample for the time period the data were collected. Thus, no cause-and-effect relationship can be drawn from the findings. Finally, the further the response rate is from 100% the greater the threat to the external validity of the findings is. However, the 69% response rate in the current study is significantly better than most published studies of nurses.

## 5. Conclusion

Nurses play a central role in the health of the public. Healthy nurse employees and a healthy work environment contribute to a workforce functioning at a high level, improved employee satisfaction, improved retention of nurse employees, improved quality of medical care, and improved patient satisfaction. The results of this study indicate that both stress and coping abilities influence the health and work performance of nurses. Worksite health promotion and disease prevention initiatives for nurses should focus on developing policies, systems, and work environments that facilitate the adoption and maintenance of healthy behaviors.

## Figures and Tables

**Table 1:** Perceptions of work-related stress and coping ability among nurses.

	*N* (%)

*In general how would you rate the level of work-related stress at this hospital?*	
Very High	4 (3.3)
High	26 (21.7)
Moderate	80 (66.7)
Low	9 (7.5)
Very Low	1 (0.8)

*How Well do You Cope with work-related stress?*	
Very well	23 (19.2)
Well	62 (51.7)
Neither well nor poorly	29 (24.2)
Poorly	5 (4.2)
Very Poorly	0 (0)

*Coping techniques used when confronted with stress *	
Eat more of favorite foods	50 (41.7)
Smoke cigarettes/tobacco use	12 (10.0)
Drink alcohol	15 (12.5)
Exercise	45 (37.5)
Talk with friends/loved ones	95 (79.2)
Sleep	26 (21.7)
Listen to music	55 (45.8)
Stay home	17 (14.2)
Cry	17 (14.2)
Play sports/participate recreation	15 (12.5)
Pray/meditate	51 (42.5)
Watch TV	51 (42.5)
Shopping	42 (35.0)
Other	22 (18.3)

**Table 2:** Coping techniques and their frequency in response to work-related stress.

Item	Every time	Majority of time	Sometimes	Rarely	Never
Eat more food than usual	2 (1.7%)	25 (20.8%)	49 (40.8%)	35 (29.2%)	9 (7.5%)
Eat less food than usual	1 (0.8%)	6 (5.0%)	27 (22.5%)	59 (49.2%)	25 (20.8%)
Eat more junk food than usual	2 (1.7%)	23 (19.2%)	59 (49.2%)	26 (21.7%)	9 (7.5%)
Eat more healthy foods than usual	0	2 (1.7%)	38 (31.7%)	56 (46.7%)	20 (16.7%)
Exercise less than usual	2 (1.7%)	12 (10.0%)	35 (29.2%)	40 (33.3%)	27 (22.5%)
Exercise more than usual	1 (0.8%)	10 (8.3%)	37 (30.8%)	44 (36.7%)	21 (17.5%)
Sleep more than usual	0	12 (10.0%)	37 (30.8%)	44 (36.7%)	27 (22.5%)
Sleep less than usual	2 (1.7%)	24 (20.0%)	47 (39.2%)	34 (28.3%)	9 (7.5%)

**Table 3:** Health status in the past month by level of stress and adequacy of coping.

Past 30 days' health status	Total population M (±SE)	Perceived stress & coping				
		High stress and poor coping M (±S.E)	High stress or poor coping M (±S.E)	Low stress and good coping M (±S.E)	*P* value	Test statistic *F*
*On how many days out of the last 30 days..*						
Did you feel healthy and full of energy	14.48 (0.90)	13.98 (1.79)	14.13 (1.11)	17.22 (2.23)	0.04^*∗*^	2.99
Did you feel like you did not get enough sleep	12.30 (0.78)	12.55 (1.06)	12.45 (1.35)	12.05 (1.35)	0.81	1.27
Did you feel worried, tense or anxious	8.48 (0.89)	18.00 (3.59)	8.98 (1.37)	6.91 (1.12)	0.01^*∗*^	6.03
Did you eat too much	8.24 (0.86)	10.22 (3.53)	9.52 (1.71)	7.27 (0.92)	0.07	1.99
Did pain make it hard for you to do your usual daily activities	3.92 (0.60)	7.40 (2.82)	4.46 (0.87)	2.16 (0.57)	0.03^*∗*^	3.36
Did you feel sad, blue or depressed	3.34 (0.52)	6.11 (2.40)	4.48 (1.14)	2.29 (0.48)	0.02^*∗*^	4.50
Did you have a poor appetite	2.22 (0.79)	3.50 (2.11)	2.72 (1.29)	1.08 (0.47)	0.05	2.07

^*∗*^Statistically significant differences in mean values across the groups measured by ANOVA at *P* < 0.05.

**Table 4:** Health behaviors of participants compared by level of stress and adequacy of coping.

Health behaviors	Yes *N* (%)	Perceived stress & coping				
		High stress and poor coping	High stress or poor coping	Low stress and good coping	*P* value	Test statistic *χ* ^2^
In the past 30 days, did you participate in any physical activities such as running, bicycling, swimming, golf or walking for the specific purpose of exercise?	93 (78%)	75%	77%	78%	0.90	1.27
Do you typically eat a minimum of 5 servings of fruits/vegetables per day?	39 (33%)	23%	33%	41%	0.06	3.93
Do you typically engage in vigorous physical activity for 20 minutes or more per day on 3 or more days per week	36 (30%)	18%	33%	39%	0.04	5.27
Do you typically engage in moderate exercise of 30 minutes or more per day for 5 or more days per week?	34 (28%)	17%	28%	31%	0.05	4.63
During the past 30 days, have you had 5 or more drinks on any occasion?	26 (22%)	24%	23%	18%	0.07	2.72
Do you typically get an average of 8 hours of sleep per night?	25 (21%)	12%	18%	28%	0.03	5.99
In the past 30 days, have you used any form of tobacco?	17 (14%)	17%	15%	13%	0.10	2.07

Statistically significant differences across the groups measured by Chi-Square tests at *P* < 0.05.

**Table 5:** Depression and anxiety symptoms score comparison by stress and coping.

PHQ-4 items score (range 0–12)	Total population M (±SE)	Perceived stress & coping			*P* value	Test statistic *F*
		High stress and poor coping	High stress or poor coping	Low stress and good coping		
Feeling nervous, anxious or on edge (range = 0–3)	1.90 (0.07)	2.58 (0.22)	1.97 (0.11)	1.75 (0.09)	0.002^*∗*^	6.39
Not being able to stop or control worrying (range = 0–3)	1.62 (0.06)	2 (0.21)	1.67 (0.14)	1.54 (0.08)	0.15	1.81
Feeling down, depressed or hopeless (range = 0–3)	1.42 (0.06)	1.75 (0.26)	1.56 (0.10)	1.29 (0.06)	0.01^*∗*^	4.63
Little interest or pleasure in doing things (range = 0–3)	1.34 (0.05)	1.58 (0.26)	1.41 (0.08)	1.27 (0.06)	0.16	1.99

^*∗*^Statistically significant differences in mean values across the groups measured by ANOVA at *P* < 0.05.

**Table 6:** Preventive health and screening behaviors in the past year comparison by stress and coping.

Within the past 1 year	*N* (%)^*ϕ*^	Perceived stress & coping			*P* value	Test statistic *χ* ^2^
		High stress and poor coping	High stress or poor coping	Low stress and good coping		
Mammogram	46 (38%)	31%	38%	67%	0.07	7.72
Pap test	72 (60%)	56%	62%	67%	0.10	5.40
Colonoscopy	11 (9%)	8%	9%	17%	0.09	6.30
Blood sugar profile	56 (47%)	37%	46%	59%	0.06	8.10
Blood lipid profile	57 (48%)	40%	46%	70%	0.04^*∗*^	11.70
Blood pressure check	112 (93%)	91%	92%	97%	0.81	3.06
General physical exam	94 (78%)	60%	77%	90%	0.03^*∗*^	12.06

^*ϕ*^ *N* (%) of those nurses who had a particular test or screening out of the total population.

^*∗*^Statistically significant differences across the groups measured by Chi-Square tests at *P* < 0.05.

**Table 7:** Situation specific self-efficacy at work compared by level of stress and adequacy of coping.

Confidence in dealing with	*N* (%)^*ϕ*^	Perceived stress & coping			Test statistic *χ* ^2^	*P* value
		High stress and poor coping	High stress or poor coping	Low stress and good coping		
Difficulties with patients	110 (92)	82%	87%	97%	5.36	0.06
Getting your work done during your shift	109 (91)	64%	87%	100%	19.72	<0.001^*∗*^
Difficulties with patient's family members	106 (88)	82%	85%	93%	2.27	0.32
Having to do a lot of tasks at the same time	101 (84)	64%	77%	94%	10.69	0.005^*∗*^
Delegating tasks to ancillary staff	101 (84)	60%	84%	91%	7.20	0.02^*∗*^
Fatigue during your shift	99 (83)	80%	82%	87%	1.80	0.62
Unclear orders from the physicians	96 (80)	73%	74%	87%	2.99	0.22
Medication errors made by others	94 (78)	70%	82%	82%	1.81	0.65
Medication errors that you make	81 (77)	60%	72%	69%	1.08	0.77
Relational difficulties with colleagues	89 (74)	55%	69%	81%	4.36	0.11
Equipment that is malfunctioning/not working	89 (74)	64%	72%	77%	1.81	0.63
Relational issues with physicians	88 (73)	27%	66%	87%	19.90	<0.001^*∗*^
Lack of timely follow-through by support staff	88 (73)	46%	67%	82%	8.10	0.01^*∗*^
Difficulties with your supervisor(s)	92 (77)	42%	81%	82%	9.52	0.009^*∗*^
Poorly defined or unclear procedures	79 (66)	54%	64%	75%	4.99	0.08

^*ϕ*^ *N* (%) of those nurses who agreed that they were “confident” or “very confident” in dealing with a particular situation or event.

^*∗*^Statistically significant differences across the groups measured by Chi-Square tests at *P* < 0.05.
